# From gut to lung: Microbiota-derived metabolites reshaping the tumor immune microenvironment

**DOI:** 10.17179/excli2026-9646

**Published:** 2026-08-20

**Authors:** Prabesh Baniya, Santosh Lamichhane, Kamal Dua, Keshav Raj Paudel

**Affiliations:** 1 Department of Biomedical Science and Engineering, Gwangju Institute of Science and Technology, Gwangju 61005, Republic of Korea; 2 Turku Bioscience Centre, University of Turku and Åbo Akademi University, Turku, 20520, Finland; 3 Research Center for Infections and Immunity, Institute of Biomedicine, University of Turku, Turku, 20520, Finland; 4 Microbe Centre, University of Turku and Wellbeing Services County of Southwest Finland, Turku, 20520, Finland; 5 Turku Clinical Microbiome Bank, Clinical Microbiology, Turku University Hospital, The Wellbeing Services County of Southwest Finland, Turku, 20520, Finland; 6 NICM Health Research Institute and School of Science, Western Sydney University, Sydney, NSW 2145, Australia

## ⁯⁯

Lung cancer remains the leading cause of cancer-related mortality worldwide, and non-small cell lung cancer (NSCLC), which comprises the vast majority of lung cancer cases, has benefited from major therapeutic progress through targeted therapies and immune checkpoint inhibitors (ICIs). Although ICIs targeting the programmed cell death protein-1 (PD-1)/programmed death-ligand 1 (PD-L1) axis have revolutionized the management of advanced NSCLC, only a subset of patients achieves durable clinical benefit, underscoring the urgent need to identify novel biomarkers and therapeutic strategies that can improve treatment efficacy. Emerging evidence indicates that the gut microbiota shapes the pulmonary tumor microenvironment, including in NSCLC (Jin et al., 2019[5]).

Beyond the microbial community itself, increasing attention has focused on gut microbiota-derived metabolites as key mediators of host-microbe communication (Yang et al., 2025[13]). These bioactive molecules regulate immune homeostasis, metabolic function, inflammatory signaling, and tumor-associated processes, making them compelling therapeutic targets for limiting lung cancer progression and enhancing the efficacy of immunotherapy. Intriguingly, this understanding is further supported by the emerging concept of the gut-lung axis. A dynamic, bidirectional communication network between anatomical distant organs that are functionally connected through shared immune, metabolic, and circulatory pathways. Metabolites produced by the gut microbiota enter systemic circulation, where they shape the phenotype and function of immune and stromal cells within the pulmonary microenvironment. Through these systemic effects, gut-derived metabolites influence tumor initiation, progression, and responsiveness to therapy, highlighting their central role in lung cancer pathogenesis and progression.

Gut microbial profiling has revealed a distinct gut microbial signature in lung cancer patients characterized by enrichment of *Bacteroides*, *Veillonella*, and *Fusobacterium* together with depletion of beneficial commensal bacteria, and these changes correlated with systemic inflammatory biomarkers (Zhang et al., 2018[15]). More recently, integrated microbiome and serum metabolomic analyses demonstrated that gut dysbiosis is accompanied by metabolic remodeling, particularly dysregulation of sphingolipid metabolism, with specific microbial taxa such as *Muribaculaceae* and *Clostridium* associated with NSCLC progression (Ni et al., 2023[9]). Collectively, these findings suggest that the functional consequences of gut dysbiosis are mediated largely through alterations in microbiota-derived metabolites, which have emerged as key regulators of antitumor immunity and tumor progression.

Among the diverse classes of gut microbiota-derived metabolites implicated in lung cancer, short-chain fatty acids (SCFAs), including acetate, propionate, and butyrate, have been the most extensively investigated owing to their well-established immunomodulatory and antitumor properties (Yang et al., 2025[13]). SCFAs are generated through the bacterial fermentation of dietary fibers, primarily by butyrate-producing commensal bacteria such as *Faecalibacterium*, *Roseburia*, and *Eubacterium*, whereas *Bifidobacterium* contributes indirectly through acetate production and metabolic cross-feeding interactions. These metabolites exert pleiotropic effects on host immunity through both receptor-dependent and epigenetic mechanisms. SCFAs activate G-protein-coupled receptors, including GPR41, GPR43, and GPR109A, while simultaneously functioning as histone deacetylase (HDAC) inhibitors, thereby regulating inflammatory signaling, immune cell differentiation, and gene expression (Fusco et al., 2023[3]). Through these molecular mechanisms, SCFAs reshape both local and systemic immune responses, creating a tumor microenvironment that is less permissive to cancer initiation, progression, and metastatic dissemination. In lung cancer, accumulating evidence demonstrates that SCFAs exert potent antitumor effects through both immune-dependent and tumor-intrinsic mechanisms. Recent evidence suggests that butyrate can suppress tumor metastasis indirectly by modulating host immunity. In a B16F10 experimental lung metastasis model, butyrate increased CCL20 expression in pulmonary endothelial cells, promoting CCR6-dependent recruitment of Th17 cells to the lungs and thereby reducing melanoma lung metastasis, without directly affecting tumor cell proliferation or apoptosis (Chen et al., 2021[2]). Furthermore, butyrate enhances the activation and metabolic reprogramming of cytotoxic CD8^+^ T cells, thereby augmenting their antitumor activity.

While SCFAs represent a well-characterized mediator of gut-lung immune crosstalk, secondary bile acids have also emerged as an important class of microbiota-derived metabolites that shape the tumor microenvironment through diverse immunoregulatory mechanisms (Wu et al., 2025[12]). Primary bile acids synthesized in the liver are converted into secondary bile acids, including deoxycholic acid (DCA), lithocholic acid (LCA), and ursodeoxycholic acid (UDCA), through bacterial biotransformation by intestinal commensals. This process is mediated predominantly by *Clostridium* spp. via 7α-dehydroxylation, with *Bacteroides* and *Lactobacillus* contributing to bile acid deconjugation and metabolism (Liu et al., 2025[6]). Beyond their role in digestion, secondary bile acids act as signaling molecules by activating receptors such as the farnesoid X receptor (FXR), Takeda G protein-coupled receptor 5 (TGR5), and vitamin D receptor (VDR), thereby regulating inflammatory signaling, epithelial barrier integrity, immune-cell differentiation, and metabolic homeostasis. Through these pathways, they influence the recruitment and function of dendritic cells, macrophages, regulatory T cells, cytotoxic CD8^+^ T cells, and natural killer cells, ultimately shaping the immune landscape of the tumor microenvironment. Importantly, the effects of secondary bile acids are highly context dependent. For example, although DCA has been reported to enhance antitumor immunity under certain conditions, chronic exposure to elevated DCA concentrations can induce oxidative stress, DNA damage, and senescence-associated secretory phenotypes that promote colorectal tumorigenesis (Cao et al., 2024[1]). In contrast, LCA and UDCA are more frequently associated with anti-inflammatory and immunoregulatory activities that support immune surveillance. Collectively, these findings suggest that the influence of secondary bile acids on cancer is determined not only by their abundance but also by the tissue context, local immune landscape, and disease stage, and that restoration of physiological bile acid homeostasis may represent a promising strategy for reinforcing antitumor immunity.

In addition to bile acids, microbial metabolism of dietary tryptophan generates a diverse array of bioactive indole derivatives that exert profound effects on immune regulation. Commensal bacteria, including *Lactobacillus*, *Bifidobacterium*, and *Peptostreptococcus*, convert tryptophan into metabolites such as indole-3-acetic acid, indole-3-propionic acid, and indole-3-aldehyde (Zelante et al., 2013[14]). These metabolites primarily activate the aryl hydrocarbon receptor (AhR), a ligand-dependent transcription factor that coordinates mucosal immunity and immune tolerance. Physiological activation of AhR by microbial indoles enhances epithelial barrier integrity, suppresses excessive inflammatory responses, regulates regulatory T-cell and type 3 innate lymphoid cell homeostasis, and promotes dendritic cell maturation. Furthermore, AhR signaling influences macrophage polarization and cytokine production, thereby limiting persistent inflammation that promotes tumor initiation and progression (Gutiérrez-Vázquez and Quintana, 2018[4]). Beyond maintaining mucosal homeostasis, recent studies indicate that microbial tryptophan metabolism also intersects with host tryptophan catabolism through the indoleamine 2,3-dioxygenase 1 (IDO1)-kynurenine pathway, a major metabolic axis exploited by tumors to establish immune tolerance. Increased IDO1 activity in the tumor microenvironment depletes local tryptophan while generating kynurenine, an endogenous AhR ligand that promotes regulatory T-cell differentiation, suppresses cytotoxic CD8^+^ T-cell and NK-cell function, and contributes to T-cell exhaustion and immune escape. In contrast, microbiota-derived indole metabolites activate AhR in a ligand-specific manner that preferentially supports epithelial homeostasis and protective immune responses, highlighting the context-dependent nature of AhR signaling. Collectively, these findings suggest that the balance between host kynurenine production and microbial indole metabolism represents a critical determinant of antitumor immunity and may provide therapeutic opportunities to reshape the tumor immune microenvironment through modulation of the gut microbiota. Another emerging microbiota-derived metabolite receiving increasing attention is inosine, a purine metabolite produced by beneficial bacteria, including *Bifidobacterium pseudolongum* and *Akkermansia muciniphila*. Emerging evidence suggests that inosine can modulate adaptive immunity in a context-dependent manner. Under specific costimulatory and metabolic conditions, inosine enhances Th1 differentiation and promotes interferon-γ production, thereby supporting CD8^+^ T cell effector functions, including activation and cytotoxicity (Mager et al., 2020[7]). These immune-modulatory effects contribute to improved antitumor immune responses in preclinical tumor models and may have relevance to lung cancer, where immune dysregulation and T cell exhaustion are key features of the tumor microenvironment.

Collectively, accumulating evidence indicates that gut microbial metabolites regulate multiple stages of the cancer-immunity cycle through complementary but interconnected mechanisms. SCFAs primarily modulate inflammatory signaling and epigenetic regulation; secondary bile acids maintain immune and metabolic homeostasis through FXR and TGR5 signaling; tryptophan-derived indoles preserve mucosal and systemic immune balance via AhR activation, whereas inosine directly enhances T-cell-mediated antitumor immunity. Together, these pathways converge on the tumor microenvironment to promote antigen presentation, facilitate dendritic cell maturation, amplify cytotoxic CD8^+^ T-cell activity, suppress chronic inflammation, and curtail the accumulation of immunosuppressive cell populations, which perhaps could be collectively reshaping the immunological landscape of NSCLC. Yet this mechanistic framework captures only a partial view, as emerging evidence reveals a far broader repertoire of microbiome-derived molecules and largely unexplored potential to bridge the gut-lung axis (Mannochio-Russo et al., 2025[8]). Beyond their classical receptor signaling roles, microbially modified bile acids are now being shown to engage novel epigenetic programs and immune-metabolic pathways whose functional significance in lung cancer remains incompletely characterized. Another emerging class of gut microbiota-derived lipid mediators, such as lysophospholipids, sphingolipids, and polyunsaturated fatty acid metabolites, is increasingly recognized as systemically active immune modulators capable of reaching the pulmonary microenvironment and influencing the immunological and stromal landscape of NSCLC. These largely unexplored lipid-based signals offer a unique opportunity to uncover novel gut-derived mechanisms that remodel the lung tumor microenvironment and reveal novel therapeutic targets for NSCLC.

The clinical relevance of this metabolite-immune axis is perhaps most strikingly illustrated in the context of immune checkpoint inhibition in NSCLC. Growing evidence suggests that patients with a metabolically favorable gut microbiome (enriched in SCFA-producing, bile acid-transforming) exhibit superior responses to PD-1/PD-L1 blockade, whereas depletion of beneficial metabolite-producing bacteria is frequently associated with primary or acquired resistance to immunotherapy (Perl et al., 2025[10]; Routy et al., 2018[11]). These observations collectively reposition the gut microbiome not as a passive bystander, but as an active, mechanistically integrated, and therapeutically tractable determinant of immunotherapy outcome in NSCLC. Critically, this repositioning opens a clinically actionable window. First, restoring a favorable microbial ecosystem and its associated metabolite output represents a biologically rational strategy to sensitize tumors to immune checkpoint blockade. To this end, multiple microbiome-targeted approaches have emerged as promising adjunctive strategies, spanning dietary fiber supplementation, prebiotic and probiotic interventions, postbiotic administration, fecal microbiota transplantation, and the deployment of engineered microbial consortia designed to maximize immunostimulatory metabolite production. Encouragingly, early-phase clinical trials are now beginning to evaluate several of these approaches in combination with PD-1/PD-L1 inhibitors, offering a first glimpse of translational feasibility. Next, the coordinated integration of high-resolution metabolomics, metagenomics, and longitudinal immune profiling within prospective clinical cohorts will be essential to define robust, metabolite-based predictive biomarkers of ICI responsiveness. In the future, harnessing the gut microbiome as a modifiable therapeutic lever through precision microbiome engineering and targeted metabolite supplementation holds transformative potential to remodel the pulmonary immune microenvironment, overcome immunotherapy resistance, and meaningfully improve clinical outcomes for patients with NSCLC.

## Declaration

### Conflict of interest

All authors have reviewed and approved the manuscript and have no conflicts of interest to declare.

### Artificial Intelligence (AI) - assisted technology

Artificial intelligence was not used in preparation of this article.

## References

[1] Cao Y, Xu Y, Zhou J, Fu X, Zhang H, Du X (2024). Farnesoid X receptor (FXR) as a potential therapeutic target for lung diseases: a narrative review. Journal of Thoracic Disease.

[2] Chen L, Zhou X, Wang Y, Wang D, Ke Y, Zeng X (2021). Propionate and Butyrate Produced by Gut Microbiota after Probiotic Supplementation Attenuate Lung Metastasis of Melanoma Cells in Mice. Mol Nutr Food Res.

[3] Fusco W, Lorenzo MB, Cintoni M, Porcari S, Rinninella E, Kaitsas F (2023). Short-chain fatty-acid-producing bacteria: key components of the human gut microbiota. Nutrients.

[4] Gutiérrez-Vázquez C, Quintana FJ (2018). Regulation of the Immune Response by the Aryl Hydrocarbon Receptor. Immunity.

[5] Jin C, Lagoudas GK, Zhao C, Bullman S, Bhutkar A, Hu B (2019). Commensal Microbiota Promote Lung Cancer Development via γδ T Cells. Cell.

[6] Liu H, Xiong X, Zhu W, Wang S, Huang W, Zhu G (2025). Gut microbial metabolites in cancer immunomodulation. Molecular Cancer.

[7] Mager LF, Burkhard R, Pett N, Cooke NCA, Brown K, Ramay H (2020). Microbiome-derived inosine modulates response to checkpoint inhibitor immunotherapy. Science.

[8] Mannochio-Russo H, Charron-Lamoureux V, van Faassen M, Lamichhane S, Gonçalves Nunes WD, Deleray V (2025). The microbiome diversifies long- to short-chain fatty acid-derived N-acyl lipids. Cell.

[9] Ni B, Kong X, Yan Y, Fu B, Zhou F, Xu S (2023). Combined analysis of gut microbiome and serum metabolomics reveals novel biomarkers in patients with early-stage non-small cell lung cancer. Frontiers in Cellular and Infection Microbiology.

[10] Perl M, Fante MA, Herfeld K, Scherer JN, Poeck H, Thiele Orberg E (2025). Microbiota-derived metabolites: Key modulators of cancer immunotherapies. Med.

[11] Routy B, Le Chatelier E, Derosa L, Duong CPM, Alou MT, Daillère R (2018). Gut microbiome influences efficacy of PD-1–based immunotherapy against epithelial tumors. Science.

[12] Wu G, Zhu L, Xue F, Zhao Y (2025). Bile acid metabolism and invasion-related genes as therapeutic monitoring biomarkers in non-small cell lung cancer. Discover Oncology.

[13] Yang Y, Ye M, Song Y, Xing W, Zhao X, Li Y (2025). Gut microbiota and SCFAs improve the treatment efficacy of chemotherapy and immunotherapy in NSCLC. npj Biofilms and Microbiomes.

[14] Zelante T, Iannitti Rossana G, Cunha C, De Luca A, Giovannini G, Pieraccini G (2013). Tryptophan Catabolites from Microbiota Engage Aryl Hydrocarbon Receptor and Balance Mucosal Reactivity via Interleukin-22. Immunity.

[15] Zhang WQ, Zhao SK, Luo JW, Dong XP, Hao YT, Li H (2018). Alterations of fecal bacterial communities in patients with lung cancer. Am J Transl Res.

